# *Fusarium mindanaoense* sp. nov., a New Fusarium Wilt Pathogen of Cavendish Banana from the Philippines Belonging to the *F. fujikuroi* Species Complex

**DOI:** 10.3390/jof9040443

**Published:** 2023-04-05

**Authors:** Shunsuke Nozawa, Yosuke Seto, Yoshiki Takata, Lalaine Albano Narreto, Reynaldo R. Valle, Keiju Okui, Shigeya Taida, Dionisio G. Alvindia, Renato G. Reyes, Kyoko Watanabe

**Affiliations:** 1 College of Agriculture, Tamagawa University, 6-1-1 Tamagawa-Gakuen, Machida, Tokyo 194-8610, Japan; nozawa@agr.tamagawa.ac.jp (S.N.);; 2 Cancer Chemotherapy Center, Japanese Foundation for Cancer Research, 3-8-31 Ariake, Koto-ku, Tokyo 135-8550, Japan; 3 Unifrutti Tropical Philippines, Inc., Km. 15, Panacan, Davao City 8000, Philippines; 4 BaCaDM Project of College of Agriculture, Tamagawa University, 6-1-1 Tamagawa-Gakuen, Machida, Tokyo 194-8610, Japan; 5 Unifrutti Japan Corporation, 1-11-1 Marunouchi, Chiyoda-Ku, Tokyo 100-6217, Japan; 6 Philippine Center for Postharvest Development and Mechanization, Science City of Muñoz 3120, Philippines; 7 Department of Biology, Central Luzon State University, Science City of Muñoz 3120, Philippines

**Keywords:** Fusarium wilt, new species, *SIX* gene, plant disease, multigene phylogeny

## Abstract

The pathogen causing Fusarium wilt in banana is reported to be *Fusarium oxysporum* f. sp. *cubense* (FOC). In 2019, wilt symptoms in banana plants (cultivar: Cavendish) in the Philippines were detected, i.e., the yellowing of the leaves and discoloration of the pseudostem and vascular tissue. The fungus isolated from the vascular tissue was found to be pathogenic to Cavendish bananas and was identified as a new species, *F. mindanaoense*, belonging to the *F. fujikuroi* species complex (FFSC); species classification was assessed using molecular phylogenetic analyses based on the *tef1*, *tub2*, *cmdA*, *rpb1*, and *rpb2* genes and morphological analyses. A reciprocal blast search using genomic data revealed that this fungus exclusively included the *Secreted in Xylem 6* (*SIX6*) gene among the *SIX* homologs related to pathogenicity; it exhibited a highly conserved amino acid sequence compared with that of species in the FFSC, but not with that of FOC. This was the first report of Fusarium wilt in Cavendish bananas caused by a species of the genus *Fusarium* other than those in the *F. oxysporum* species complex.

## 1. Introduction

Bananas are one of the most common agricultural exports, though they are also widely consumed in the countries that produce them [1]. *Musa sapientum* cv. Cavendish (AAA group) is exclusively cultivated in many tropical countries as a commercial crop; 21 million tons of Cavendish bananas were exported in 2019 (FAO 2022). In the 1950s, the planting of Cavendish bananas instead of cv. Gros Michel began to increase worldwide because of an epidemic of Fusarium wilt disease (Panama disease) caused by *Fusarium oxysporum* f. sp. *cubence* (FOC) race 1. Thereafter, the causal pathogens of Fusarium wilt disease in bananas were found and characterized as race 1, race 2, race 4, subtropical race 4 (STR4), and tropical race 4 (TR4) based on their pathogenicity. In the 1990s, TR4 was identified as the causal agent of Fusarium wilt disease infecting Cavendish bananas in Taiwan [2]. The disease caused by TR4 has been reported in 23 countries (predominantly in Southeast Asia, South Asia, Africa, and Latin America) [3]. Moreover, TR4 affected the banana industry and reduced banana yield in the Philippines (FAO 2022). Mostert et al. [4] and Solpot et al. [5] investigated the Fusarium wilt pathogen in the Philippines; mostly TR4 (VCG01223/16), and less commonly R4 (VCG0122), was detected in Mindanao. However, information on FOC other than TR4 is scarce and no reports of other *Fusarium* species are available.

**Figure 1:**
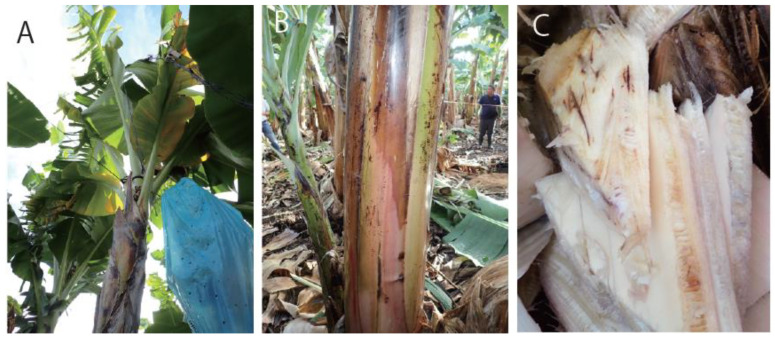
Infection symptoms of bananas (cv. Cavendish). (**A**) yellowish leaves; (**B**) brownish pseudostem; (**C**) brownish xylem.

In this study, during a survey of the Fusarium wilt disease in Mindanao conducted in September 2019, a new species was found that belonged to the *F. fujikuroi* species complex (FFSC); it caused symptoms of leaf yellowing (Figure 1A) to emerge in older leaves and a reddish-brown discoloration of the pseudostem and vascular tissues of bananas (Cavendish) (Figure 1B,C). This pathogen had not been previously reported to cause Fusarium wilt in banana. Therefore, we aimed to identify this causal agent, conducted molecular and morphological analyses, and proposed an isolate as a new pathogenic species of Fusarium wilt.

Furthermore, to provide fundamental information relating to factors of its pathogenicity, we searched the *Secreted in Xylem* (*SIX*) genes from the whole-genome data of the new species. In addition, we predicted whether the genome of the new *Fusarium* species obtained *SIX* genes via horizontal transfer from FOC or by other means; it is reported that *SIX* genes are one of the most important factors for infecting banana plants [6,7,8].

## 2. Materials and Methods

### 2.1. Sample Collection and Fungal Isolation

Symptomatic banana plants were collected from a farm in Mindanao in 2019. The discolored vascular tissues (Figure 1C) of the pseudostem were cut into pieces of approximately 3 mm^2^, which were then sterilized with 0.6% (*v*/*v*) sodium hypochlorite for 1 min, washed with sterilized water, dried with sterilized paper, and placed on a water agar (WA) plate. The hyphae that emerged on WA were transferred onto a potato dextrose agar (PDA) plate to produce conidia for monoculture. The PD20-05 isolate was maintained on a PDA plate.

### 2.2. Genomic DNA Extraction

DNA was extracted from the mycelia of each isolate, which were grown for 7–10 days in yeast glucose medium using the modified CTAB method [9]. After treatment with chloroform–isoamyl alcohol (24:1), 2-Mercaptoethanol and 10% CTAB at 0.2% and 2%, respectively, were added to the supernatant and incubated for 40 min at 60 °C. After incubation, an equal volume of chloroform–isoamyl alcohol (24:1) was added, mixed gently for 10 min, and centrifuged for 10 min at 12,000 rpm for purification. The aqueous phase was carried out, and the above-mentioned purification was again conducted. Precipitation was achieved by adding 2.5 and 0.1 times the volume of ethanol and 3 M sodium acetate, respectively, which was then mixed for a short period of time and centrifuged for 10 min at 12,000 rpm. After removing the liquid, a pellet of DNA at the bottom was dried and dissolved with 30 µL of TE buffer.

### 2.3. Gene Prediction

Genome DNA was sequenced using the Illumina HiSeq genome analyzer platform and DNA libraries and paired-end (PE) genomic libraries were generated. The libraries were sequenced in PE mode with 150 bp reads on the Illumina HiSeq X instrument. Adaptors were eliminated from reads using the Trimmomatic read trimming tool for Illumina NGS data, with a quality cut-off of 30. The raw mate–pair read sequence quality was checked using FastQC vers. 0.11.8 [10] (http://www.bioinformatics.babraham.ac.uk/projects/fastqc accessed on 12 December 2022). Platanus allee vers. 2.0.2 [11] was used to assemble the reads and obtain contig data. The N50 values were calculated to measure the quality of the assemblies. Augustus 3.3.3 [12] was used to perform gene predictions using *F. graminearum* data as a reference.

### 2.4. Phylogenetic Analyses

Molecular analyses were conducted to identify the pathogen. To select the DNA sequences, the translation elongation factor 1-alpha (*tef1*), beta-tubulin (*tub2*), calmodulin (*cmdA*), RNA polymerase large subunit (*rpb1*), and RNA polymerase second-largest subunit (*rpb2*) genes were amplified according to the method reported by Yilmaz et al. [13]; the genes were then sequenced using the following primer pairs: EF1 and EF2 [14], T1 and T2 [15], CL1 and CL2A [16], Fa [17] and R8 [18], and 5F2 [19] and 7cr [20], respectively. The sequence data were deposited in the DNA Data Bank of Japan (Table 1). One hundred and three sequences (Table 1) of each DNA region were aligned using Clustal W in MEGA 7 [21], concatenated, and subjected to phylogenetic analyses using the maximum-likelihood (ML), maximum-parsimony (MP), and neighbor-joining (NJ) methods. The reliability of the branches on the phylogenetic tree was evaluated using the bootstrap (BS) [22] test with 1000 replicates.

### 2.5. Morphological Analyses

Mycelial plugs (φ7 mm) of the isolate were placed in the center of the potato dextrose agar (PDA), synthetic nutrient-poor agar (SNA) [23], and oatmeal agar (OA) [24] plates and incubated for 6 days at 25 °C in the dark. The colony character on the surface and reverse sides was observed. The isolate (PD20-05) was cultured on carnation leaf agar (CLA) [25] and SNA, inducing sporodochial conidia and microconidia to observe its asexual morphological characteristics. Thirty conidia and conidiophores were observed under a light microscope (BX51, Olympus, Tokyo, Japan) to record their shape and size. For the mycelial growth test, 6 d cultures of the isolate grown at 25 °C on PDA plates were used. Mycelial plugs (φ7 mm) were then placed on the center of the PDA plates. These plates were incubated at 4 °C, 10 °C, 15 °C, 20 °C, 25 °C, 30 °C, and 35 °C in the dark. After incubation for 6 days, mycelial growth per day was calculated. The average growth rate per day for each temperature was determined from five replicates.

### 2.6. Pathogenicity Test

A pathogenicity test was conducted using a conidial suspension in sterilized water adjusted to 1 × 10^7^ conidia/mL. Six Cavendish seedlings were used in this experiment, in which the roots of three seedlings were soaked in 500 mL of the conidial suspension for 3 h, before being planted in pots with a 1:1 mixture of red ball earth and humus. A 5 g/L solution of NPK 8-8-8 was added as a chemical fertilizer. The remaining three seedlings were treated with sterilized water as a control. The treated and control plants were inoculated for 34 days at 25 °C with an 8 h light/16 h dark cycle.

### 2.7. Detection of Secreted in Xylem Genes among the Whole-Genome Data

A tBLASTn analysis was conducted to identify the *SIX* genes using BLAST 2.11.0+ software [26,27]. In this analysis, previously reported *SIX* gene protein sequences were obtained from the NCBI protein database and were used as queries against the assembled whole-genome sequence (*e*-value = 0.001). The identified new *SIX* gene sequence was reciprocally BLAST searched (BLASTP) against the NCBI-deposited protein sequences of *SIX* genes to estimate their sequence similarity. The SignalP program (v. 5.0) [28] was used to identify the new SIX protein code signal peptides.

### 2.8. Identification of the Homologous SIX Genes of PD20-05 in the Foc TR4 Genome

To search for the homologous *SIX* genes of PD20-05 in *Fusarium oxysporum* f. sp. *cubense* (FOC) TR4, we initially constructed the genome sequence of FOC TR4 using the NGS data (SRR10054447) [29] and Platanus-allee. Subsequently, the *SIX* gene of PD20-05 was used as a query against the assembled FOC TR4 genome sequence (*e*-value ≤ 0.001).

To clarify whether the *SIX6* gene identified in our isolate was from FOC, the putative *SIX6* gene protein sequences of our isolate and those of FFSC and FOC were aligned using clustalW in MEGA7 [21], followed by the construction of an NJ tree with the option to completely delete the gap. The reliability of the branches of the phylogenetic tree was evaluated via the BS test [22] with 1000 replicates.

## 3. Results

### 3.1. Phylogenetic Analysis

**Figure 2:**
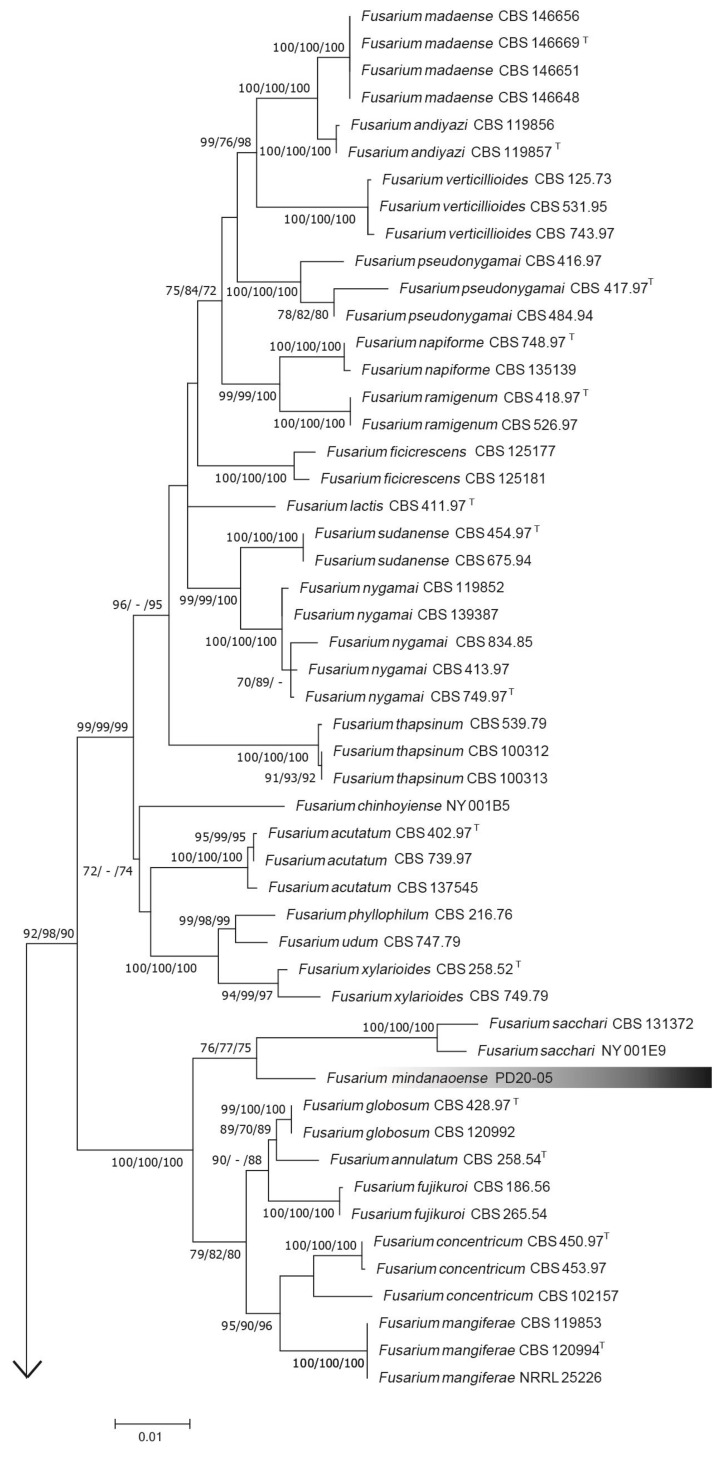

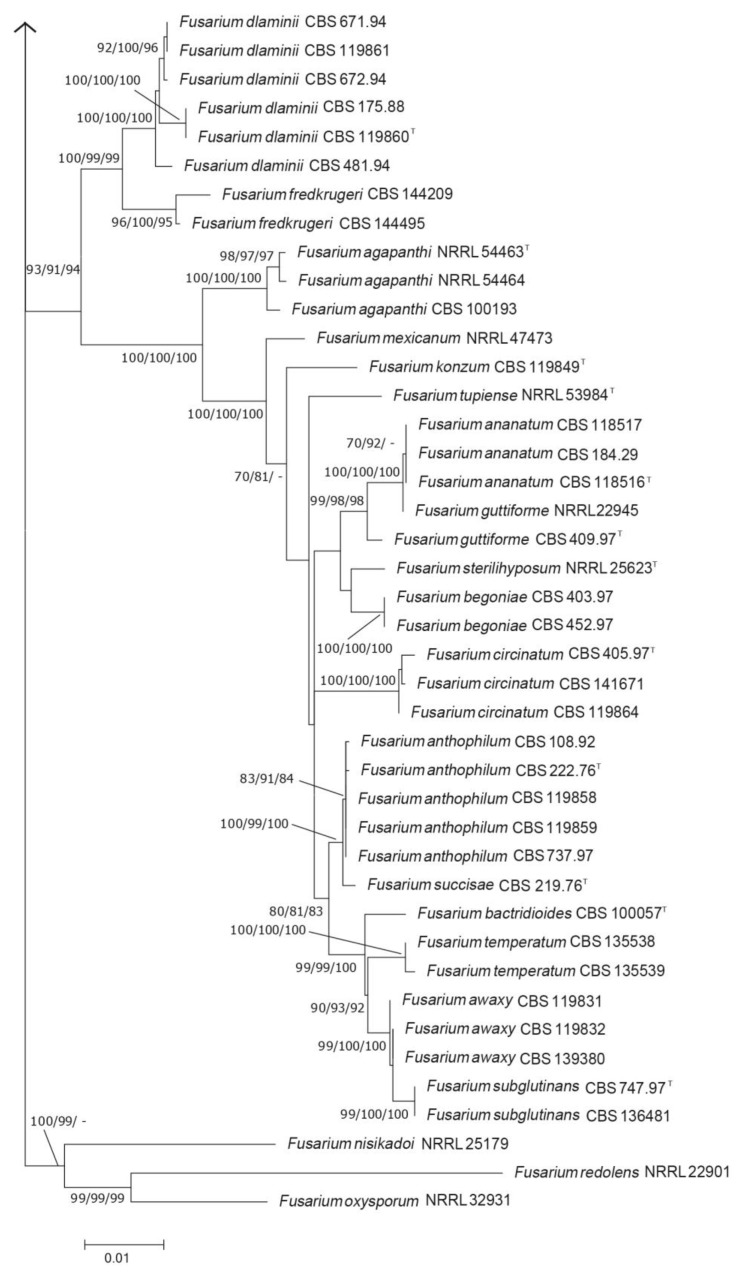
Maximum likelihood (ML) tree based on combined data sets of *tef1α*, *tub2*, *cmdA*, *rpb1*, and *rpb2* sequences. ML, maximum-parsimony (MP), and neighbor-joining (NJ) bootstrap values are indicated at the nodes as ML/MP/NJ. The hyphen (“-”) indicates that a node is not present. “^T^” indicates the ex-type and ex-epitype strains.

For the phylogenetic analysis using the five loci, the final dataset included 2606 positions (excluding gaps and including sites), comprising 462, 280, 521, 594, and 749 positions from *tef1*, *tub2*, *cmdA*, *rpb1*, and *rpb2* gene sequences, respectively. The PD20-05 isolate was independent of known species and a sister lineage of the *F. sacchari* clade (BS value = 76; Figure 2).

### 3.2. Taxonomy

**Figure 3:**
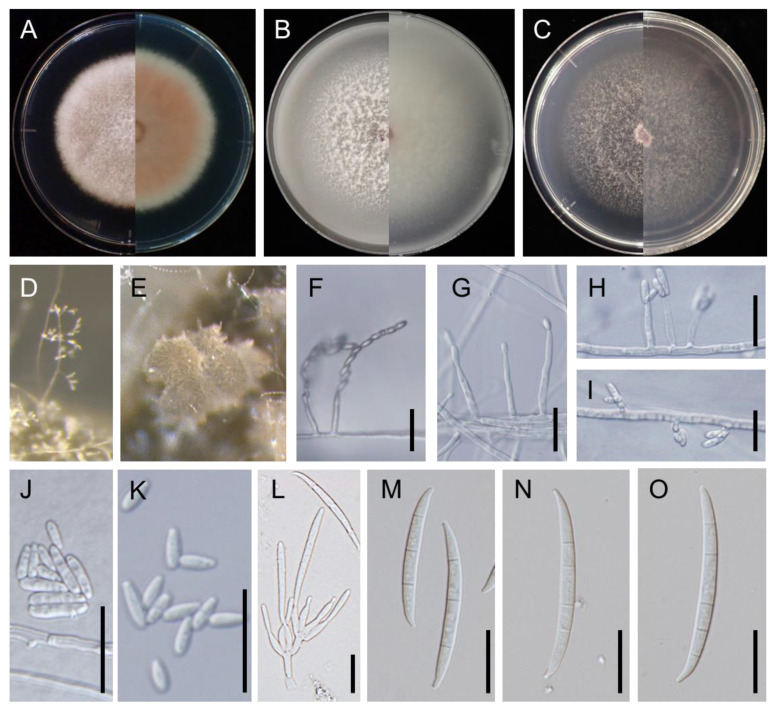
Colony morphology of *F. mindanaoense* (PD20-05T; ex-type culture PD20-05) after 6 days growth at 25 °C in the dark: (**A**). PDA; (**B**). OA; (**C**). SNA. Colony surface is shown on left half of each plate and colony undersurface on right half. (**D**). Conidiophore on carnation leaf; (**E**). sporodochia on carnation leaf; (**F**,**G**). microconidia on a conidiophore on aerial hyphae on SNA; (**H**,**I**). microconidia on a conidiophore on hyphae inside SNA; (**J**). microconidia on a carnation leaf; (**K**). microconidia on SNA; (**L**). conidiophores and phialides on sporodochia; (**M**–**O**). sporodochial conidia (macroconidia): (**M**). 3-septate conidia; (**N**). 4-septate conidia; (**O**). 5-septaate conidia. Scale bars: 20 μm.

*Fusarium mindanaoense* Nozawa & Watanabe, sp. nov.
Mycobank MB 848129; Figure 3.
Etymology: the name refers to Mindanao, the region where the ex-type strain was obtained.
Holotype: PD20-05S.
Ex-holotype: PD20-05.

**Figure 4:**
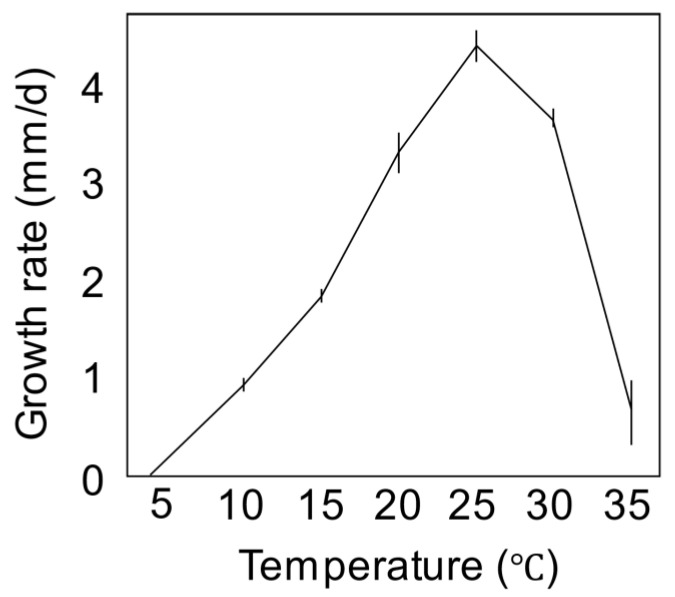
Mycelial growth rate of *F. mindanaoense* PD20-05 on PDA depending on the temperature in the dark for 6 days.

Colonies on PDA reached 50–54 mm diam. after 6 d at 25 °C in the dark; the colonies were raised, aerial mycelia dense, covered the entire margin and surface in the center, and were white at the edge. Colonies were also reverse pink in the center and white at the margin. Hyphae grew at 10–35 °C, with an optimum temperature of 25 °C (avg. ± sd. 4.4 ± 0.16 mm/day; Figure 4). Colonies on OA reached 66–68 mm diam. after 6 d at 25 °C in the dark were raised, aerial mycelia dense, and covered colony margin entire; they were also surface white and reverse white. Colonies on SNA reached 59–61 mm diam. after 6 days at 25 °C in the dark; these colonies were raised, aerial mycelia sparse, covered the entire colony margin entire, and were surface white and reverse white.

Sporodochia milk white formed on carnation leaves deficiently. Conidiophores in sporodochia were verticillately branched; bearing apical pairs were monophialide, while sporodochial phialides subulate to subcylindrical. Sporodochial macroconidia falcate were moderately curved and slender with parallel side tapering slightly toward both ends, as well as being papillate, 3–5-septate, hyaline, thin- and smooth-walled. The 3-septate conidia had dimensions of 37.7–52.6 × 3.5–5.0 (av. ± sd. 45.9 ± 3.8 × 4.2 ± 0.35) µm, while 4-septate conidia had dimensions of 50.4–66.4 × 3.1–4.6 (av. ± sd. 57.6 ± 3.4 × 3.7± 0.39) µm; 5-septate conidia had dimensions of 55.5–67.8 × 3.2–4.1 (av. ± sd. 62.1 ± 4.3 × 4.1 ± 0.7) µm. Conidiophores borne on aerial mycelia on carnation leaf were branched, while those borne on aerial mycelia SNA, bearing chained microconidia, or with values of 15.7–42 (av. ± sd. 24.2 ± 7) μm tall were either unbranched or rarely branched, instead bearing terminal monophialide. Those borne inside SNA were 0–24 (av. ± sd. 8 ± 7.5) µm tall and unbranched; they were had microconidia hyaline, oval, pyriform, smooth- and thin-walled aseptate. The microconidia on carnation leaf was 6.6–13 × 2–3.4 (av. ± sd. 8.6 ± 1.5 × 2.7 ± 0.38) µm, while microconidia on SNA was 7.6–15.8 × 2.2–3.8 (av. ± sd. 10.1 ± 2 × 2.9 ± 0.39) µm. Chlamydospores were not observed.

Note: *F. mindanaoense* resembled *F. concentricum* regarding the size of sporodochial conidia (Table 2). However, *F. mindanaoense* could be distinguished by the characteristics of colonies. *F. mindanaoense* did not produce concentric aerial hyphae in its mycelium (Figure 3A), while *F. concentricum* did produce this symptom of fungal infection. A holotype and ex-holotype strain were deposited at Flora and Fauna Analytical and Diagnostic Center at Central Luzon State University.

### 3.3. Pathogenicity Test

**Figure 5:**
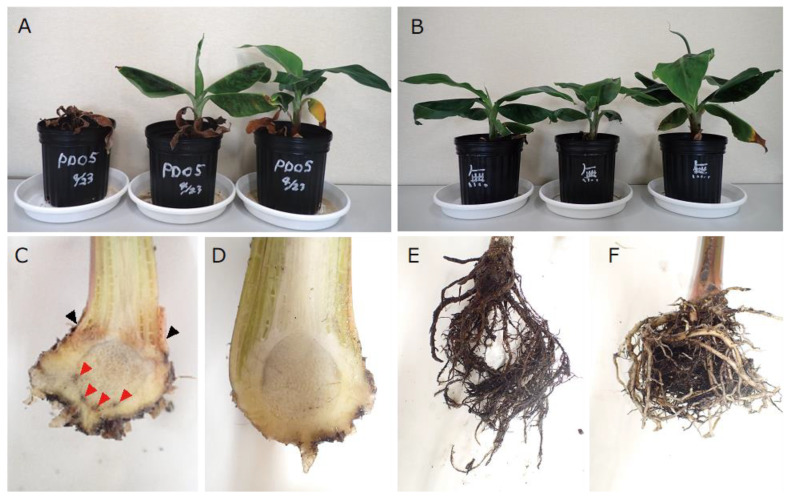
Pathogenicity test of *F. mindanaoense* PD20-05 using bananas (cv. dwarf Cavendish). (**A**). Wilting symptoms 34 days after inoculation with *F. mindanaoense* PD20-05. (**B**). Control plants without inoculation with *F. mindanaoense* PD20-05. (**C**). A tuber of the inoculated plant with blackish tissues (red arrows) and discolored tissues (black arrows). (**D**). A tuber of the control plant. (**E**). The roots of the inoculated plant. (**F**). The roots of the control plant.

Yellow leaves appeared on inoculated plants after 20–34 days; one dried-up seedling and leaves of other seedlings closed around the main veins (Figure 5A,B). Part of the internal tissues of the corms turned black, while the tissues of pseudostem just above the corn were reddish-brown (Figure 5C,D). Additionally, the roots turned black all around (Figure 5E,F). The inoculated strain was re-isolated from the discolored roots and vascular lesions, whereas the control plants treated with water exhibited no symptoms.

### 3.4. Detection of Secreted in Xylem Genes in Whole-Genome Data

**Figure 6:**
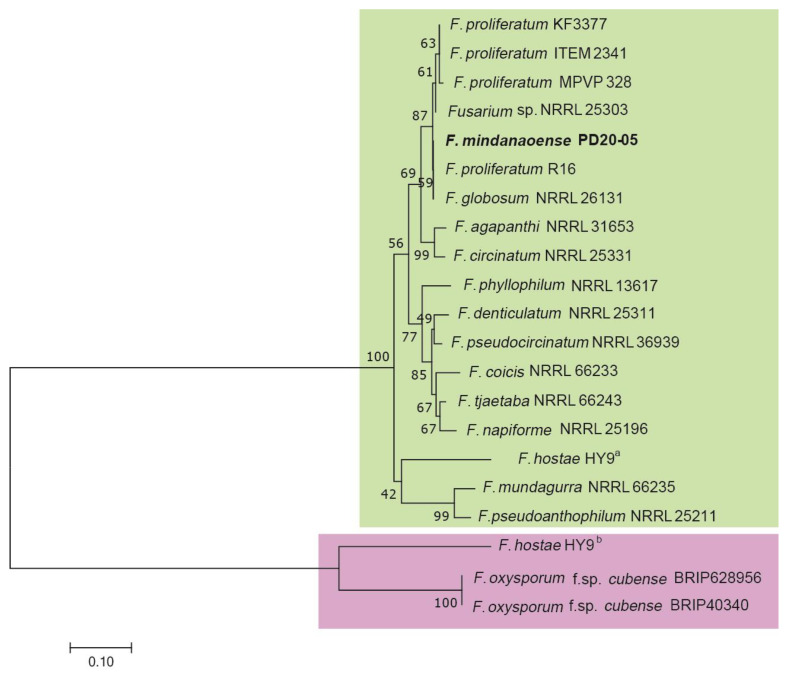
NJ phylogenetic tree based on SIX6 gene sequences. The genes highlighted in red are the SIX6 genes of FFSC, whereas the genes highlighted in green are the *SIX6* genes of FOC and *F. hostae*. *F. hostae* has two types of *SIX6* genes. One belongs to FFSC type (^a^), and another one belongs to FOC type (^b^).

In this study, the genomic DNA of PD20-05 was sequenced and assembled into 3377 contigs with an N50 of 53.7 kb and a maximum length of 164.3 kb. In the tBLASTn analysis, *SIX* gene sequences were searched in the PD20-05 genome using the 1186 NCBI-deposited SIX protein sequences. The analysis showed that the *SIX6* gene was the only SIX gene found in the PD20-05 genome sequence. Moreover, the SIX6 protein of PD20-05 was predicted to contain signal peptides (Figure S1). Furthermore, a reciprocal BLASTp analysis showed that the SIX6 protein sequence of PD20-05 was identical to that of *Fusarium* sp. NRRL 25303, *F. proliferatum*, *F. globosum*, *F. agapanthi*, *F. denticulatum*, *F. tjaetaba*, *F. napiforme*, *F. pseudocircinatum*, *F. circinatum*, *F. phyllophilum*, *F. mundagurra*, and *F. pseudoanthophilum* (Table 3), which all belong to the FFSC species and made one clade in the phylogenetic tree (Figure 6). These *SIX6* gene sequences were greatly different from those of two FOC strains (accession nos. KX435007 and KX435008) [8], as assessed based on the alignment (Figure S1).

Two types of *SIX6* genes in the *F. hostae* (HY9) genome were obtained by conducting a BLASTp using the SIX6 gene sequences of FOC (BRIP628956) and *F. mindanaoense* (PD20-05) as query sequences with low *e*-values (3 × 10^−82^ and 2 × 10^−120^, respectively). Van Dam and Rep [33] reported that the strain acquired one *SIX6* gene via horizontal transfer from the FOC. The two types of *SIX6* genes fell into different clades in the phylogenetic tree (Figure 6).

## 4. Discussion

*Fusarium sacchari* (leaf blight on AAA genome group and fruit rot on AAA), *F. proliferatum* (fruit rot on AAB and sheath rot on ABB), *F. fujikuroi* (fruit rot on AA), *F. concentricum* (fruit rot on AAA), *F. verticillioides* (fruit rot on *Musa* sp.), and *F. musae* (fruit rot on *Musa* sp.) belonging to FFSC were reported as banana pathogens [34,35,36,37,38,39,40,41]. These species do not cause Fusarium wilt of bananas. Maryani et al. [39] also isolated *F. proliferatum* from a symptomatic tissue of Fusarium wilt of banana (AA) in 2019, concluding that the fungus was not a pathogen of Fusarium wilt of banana (Cavendish: AAA); rather, it was an endophyte because it caused only a slight discoloration in the corm without any further disease development. Moreover, in 2022, Thi et al. [42] also isolated FFSC species (*F. fujikuroi*) from symptomatic tissues of Fusarium wilt of banana (ABB). However, a pathogenicity assay was not carried out. To the best of our knowledge, no FFSC species have been reported as the pathogen underlying Fusarium wilt in banana. In this study, we identified a new causal agent, *F. mindanaoense* (which belongs to FFSC), of Fusarium wilt in banana in the Philippines. This is the first report of Fusarium wilt in banana caused by a fungus belonging to the FFSC.

As FOC affects Cavendish bananas, research on FOC has focused on managing Fusarium wilt disease. Therefore, rapid detection methods for FOC, such as loop-mediated isothermal amplification and PCR detection, have been developed for diagnosis and occurrence monitoring [43,44,45]. Our study reveals that a pathogen belonging to the FFSC also caused Fusarium wilt in the Cavendish banana. Focusing on FOC and other pathogenetic fungi to acquire basic knowledge that may contribute to controlling Fusarium wilt is necessary.

The *SIX* genes play a role in the pathogenicity of Fusarium wilt; *SIX1*, *SIX2*, *SIX6*, *SIX7*, *SIX9G1*, *SIX11*, and *SIX13* were detected in the FFSC species [34,37]. The present study showed that the *F. mindanaoense* genome possessed the *SIX6* gene exclusively, which matched with those of the FFSC with low *e*-values (Table 2; 0–3.92 × 10^−125^). Van Dam and Rep [33] reported that the *SIX6* gene from *F. hostae* (HY9), which belongs to the FFSC species, was horizontally transferred from FOC. We found that *F. hostae* (HY9) has two types of *SIX6* genes: the FOC and FFSC groups (Figure 6). Because the gene sequence of *F. mindanaoense* that was identified as the *SIX6* gene did not belong to a clade of FOC, and one of the *SIX6* genes obtained from *F. hostae* genome belonged to the FFSC in the phylogenetic tree, *F. mindanaoense* was thought not to have acquired its pathogenicity through horizontal gene transfer from FOC (Figure 6). However, a functional analysis of the *SIX6* gene of the FFSC is warranted to clarify whether the *SIX6* gene acts as a functional gene in the pathogenicity of Fusarium wilt.

## Figures and Tables

**Table 1:** Strains of the *Fusarium fujikuroi* species complex used in this study with GenBank accession number.

Species	Culture Collections	GenBank Accession Number				
		*tef1*	*tub2*	*cmdA*	*rpb2*	*rpb1*
*F. acutatum*	CBS 402.97 ^T^	MW402125	MW402323	MW402459	MW402768	MW402653
	CBS 739.97	AF160276	MW402348	AF158329	MN193883	MW402696
	CBS 137545	MN533987	MN534062	MN534147	MN534228	MW402587
*F. agapanthi*	CBS 100193	MW401959	MW402160	MW402363	MW402727	MW402491
	NRRL 54463 ^T^	KU900630	KU900635	KU900611	KU900625	KU900620
	NRRL 54464	MN193856	KU900637	KU900613	KU900627	MW402718
*F. ananatum*	CBS 118516 ^T^	LT996091	MN534089	MW402376	LT996137	MW402507
	CBS 118517	MN533988	MN534090	MN534157	MN534229	MW402508
	CBS 184.29	MW402105	MW402303	MW402445	MW402809	MW402629
*F. andiyazi*	CBS 119856	MN533989	MN534081	MN534174	MN534286	MW402523
	CBS 119857 ^T^	MN193854	LT996113	MN534175	LT996138	MW402524
*F. annulatum*	CBS 258.54 ^T^	MT010994	MT011041	MT010908	MT010983	MT010944
*F. anthophilum*	CBS 108.92	MW401965	MW402166	MW402368	MW402783	MW402498
	CBS 119858	MN533990	MN534091	MN534158	MN534232	MW402525
	CBS 119859	MN533991	MN534092	MN534164	MN534233	MW402526
	CBS 222.76 ^ET^	MW402114	MW402312	MW402451	MW402811	MW402641
	CBS 737.97	MN533992	MN534093	MN534160	MN534234	MW402695
*F. awaxy*	CBS 119831	MN534056	MN534108	MN534167	MN534237	MW402514
	CBS 119832	MN534057	MN534106	MN534170	MN534240	MW402515
	CBS 139380	MN534058	MN534107	MN534172	MN534238	MW402597
*F. bactridioides*	CBS 100057 ^T^	MN533993	MN534112	MN534173	MN534235	MW402490
*F. begoniae*	CBS 403.97	MN193858	U61543	MW402460	MN193886	MW402654
	CBS 452.97 ^T^	MN533994	MN534101	MN534163	MN534243	MW402675
*F. chinhoyiense*	NY 001B5	MN534051	MN534083	MN534197	MN534263	MW402725
*F. circinatum*	CBS 405.97 ^T^	MN533997	MN534097	MN534199	MN534252	MW402656
	CBS 119864	MW401996	MW402196	MW402389	MW402736	MW402528
	CBS 141671	MW402083	MW402282	MW402427	MW402807	MW402610
*F. concentricum*	CBS 450.97 ^T^	AF160282	MW402334	MW402467	JF741086	MW402674
	CBS 453.97	MN533998	MN534123	MN534216	MN534264	MW402676
	CBS 102157	MW401963	MW402164	MW402367	MW402728	MW402496
*F. dlaminii*	CBS 175.88	MN534002	MN534138	MN534150	MN534256	MW402623
	CBS 481.94	MN534003	MN534139	MN534151	MN534257	MW402679
	CBS 671.94	MN534004	MN534136	MN534152	MN534254	MW402690
	CBS 672.94	MN534005	MN534137	MN534153	MN534255	MW402691
	CBS 119860 ^T^	MW401995	MW402195	MW402388	KU171701	KU171681
	CBS 119861	MN534001	MN534135	MN534149	MN534253	MW402527
*F. ficicrescens*	CBS 125177	MN534006	MN534071	MN534176	MN534281	MW402545
	CBS 125181	MN534007	MN534072	MN534177	MN534282	MW402548
*F. fredkrugeri*	CBS 144209 ^T^	LT996097	LT996118	LT996181	LT996147	LT996199
	CBS 144495	LT996096	LT996117	LT996180	LT996146	LT996198
*F. fujikuroi*	CBS 186.56	MW402108	MW402306	MW402447	MW402765	MW402632
	CBS 265.54	MN534011	MN534132	MN534222	MN534268	MW402650
*F. globosum*	CBS 428.97 ^T^	KF466417	MN534124	MN534218	KF466406	MW402668
	CBS 120992	MW401998	MW402198	MW402390	MW402788	MW402529
*F. guttiforme*	CBS 409.97 ^T^	MT010999	MT011048	MT010901	MT010967	MT010938
	NRRL 22945	AF160297	U34420	AF158350	JX171618	JX171505
*F. konzum*	CBS 119849 ^T^	LT996098	MN534095	LT996182	MW402733	MW402519
*F. lactis*	CBS 411.97 ^ET^	MN193862	MN534077	MN534178	MN534275	MW402659
*F. madaense*	CBS 146648	MW402095	MW402294	MW402436	MW402761	MW402616
	CBS 146651	MW402096	MW402295	MW402437	MW402762	MW402617
	CBS 146656	MW402097	MW402296	MW402438	MW402763	MW402618
	CBS 146669 ^T^	MW402098	MW402297	MW402439	MW402764	MW402619
*F. mangiferae*	CBS 119853	MN534016	MN534140	MN534225	MN534270	MW402522
	CBS 120994 ^T^	MN534017	MN534128	MN534224	MN534271	MW402530
	NRRL 25226	AF160281	U61561	AF158334	HM068353	MW402712
*F. mexicanum*	NRRL 47473	GU737416	GU737308	GU737389	LR792615	LR792579
*F. mindanaoense* (this study)	**PD20-05**	**LC720609**	**LC720611**	**LC720610**	**LC720608**	**LC720612**
*F. napiforme*	CBS 748.97 ^T^	MN193863	MN534085	MN534192	MN534291	MW402701
	CBS 135139	MN534019	MN534084	MN534183	MN534290	MW402572
*F. nygamai*	CBS 413.97	MW402127	MW402325	MW402462	MW402815	MW402660
	CBS 749.97 ^T^	MW402151	MW402352	MW402479	EF470114	MW402703
	CBS 834.85	MW402154	MW402355	MW402482	MW402821	MW402707
	CBS 119852	MW401992	MW402192	MW402386	MW402734	MW402521
	CBS 139387	MW402073	MW402272	MW402419	MW402753	MW402601
*F. phyllophilum*	CBS 216.76 ^T^	MN193864	KF466443	KF466333	KF466410	MW402637
*F. pseudonygamai*	CBS 416.97	MN534030	MN534064	MN534194	MN534283	MW402663
	CBS 417.97 ^T^	AF160263	MN534066	AF158316	MN534285	MW402664
	CBS 484.94	MN534031	MN534065	MN534195	MN534284	MW402681
*F. ramigenum*	CBS 418.97 ^T^	KF466423	MN534145	MN534187	KF466412	MW402665
	CBS 526.97	MN534032	MN534086	MN534188	MN534292	MW402682
*F. sacchari*	CBS 131372	MN534033	MN534134	MN534226	MN534293	MW402560
	NY 001E9	MN534034	MN534133	MN534227	MN534294	MW402726
*F. sterilihyposum*	NRRL 25623 ^T^	MN193869	AF160316	AF158353	MN193897	MW402713
*F. subglutinans*	CBS 747.97 ^NT^	MW402150	MW402351	MW402478	MW402773	MW402700
	CBS 136481	MW402059	MW402258	MW402413	MW402748	MW402585
*F. succisae*	CBS 219.76 ^ET^	AF160291	U34419	AF158344	MW402766	MW402639
*F. sudanense*	CBS 454.97 ^T^	MN534037	MN534073	MN534179	MN534278	MW402677
	CBS 675.94	MN534038	MN534074	MN534182	MN534279	MW402693
*F. temperatum*	CBS 135538	MN534039	MN534111	MN534168	MN534239	MW402575
	CBS 135539	MN534040	MN534110	MN534169	MN534242	MW402576
*F. thapsinum*	CBS 539.79	MW402140	MW402340	MW402472	MW402818	MW402686
	CBS 100312	MW401961	MW402162	MW402365	MW402780	MW402494
*F. thapsinum*	CBS 100313	MW401962	MW402163	MW402366	MW402781	MW402495
*F. tupiense*	NRRL 53984 ^T^	GU737404	GU737296	GU737377	LR792619	LR792583
*F. udum*	CBS 747.79	MN193872	MN534141	MN534154	MN534258	MW402699
*F. verticillioides*	CBS 125.73	MW402012	MW402212	MW402392	MW402791	MW402543
	CBS 531.95	MW402136	MW402336	MW402468	MW402771	MW402683
	CBS 734.97	MW402146	MW402346	AF158315	EF470122	MW402694
*F. xylarioides*	CBS 258.52 ^T^	MN193874	AY707118	MW402455	HM068355	MW402646
	CBS 749.79	MN534049	MN534143	AF158326	MN534259	MW402702

^T^ Ex-type specimen. ^ET^ Ex-epitype specimen. ^NT^ Ex-neotype specimen. The sequences deposited to GenBank in this study are shown in bold.

**Table 2:** Comparison of the size, septation, and shape of sporodochial conidia among related species of FFSC.

Species	Size (µm)	Septate	Shape	Substrate/ Media	References
*Fusarium mindanaoense*	37.7–52.6 × 3.5–5.0 (av. ± sd. 45.9 ± 3.8 × 4.2 ± 0.35; 3-septate) 50.4–66.4 × 3.1–4.6 (av. ± sd. 57.6 ± 3.4 × 3.7 ± 0.39; 4-septate) 55.5–67.8 × 3.2–4.1 (av. ± sd. 62.1 ± 4.3 × 4.1 ± 0.7; 5-septate)	3-5	Slightly curved	CLA	This study
*F. annulatum*	13–58 × 1.9–3.3	3-6	Menidiform or annular	Not mentioned	Bugnicourt [30]
*F. concentricum*	53.5–61.4 × 3.7–4 (avg. 57.4 × 3.7)	3-5	Slightly curved	SNA	Nirenberg and O’Donnell [23]
*F. mangiferae*	43.1–61.4 × 3 1.9–3.4 (avg. 51.8 × 2.3)	3-5	Slightly curved	CLA	Britz et al. [31]
*F. sacchari*	35.5–49.5 × 3.3–4.1	1-5	Slightly curved	SNA	Nirenberg [32]

**Table 3:** Results of the BLASTp analysis using predicted *SIX6* of PD20-05.

Hit_Defintion	Score	*e*-Value	Query_from	Query_to	Hit_from	Hit_to	Identity
KAF5645217.1 secreted in xylem *Fusarium* sp. NRRL 25303	504.597	0	1	246	1	246	241
KAG4288609.1 secreted in xylem 6 *Fusarium proliferatum*	503.056	0	1	247	1	247	240
KAG4277728.1 secreted in xylem 6 *Fusarium proliferatum*	501.13	0	1	247	1	247	240
KAG4252980.1 secreted in xylem 6 *Fusarium proliferatum*	499.204	0	9	247	1	239	239
RBA12867.1 secreted in xylem 6 *Fusarium proliferatum*	498.049	0	1	247	1	247	239
KAF5709672.1 secreted in xylem *Fusarium globosum*	498.049	0	9	246	1	238	238
KAF4501124.1 secreted in xylem 6 *Fusarium agapanthi*	484.567	3.32 × 10^−178^	1	246	1	246	230
KAF5689079.1 secreted in xylem *Fusarium denticulatum*	476.093	6.97 × 10^−175^	1	246	1	246	224
KAF5626692.1 secreted in xylem 6 *Fusarium tjaetaba*	475.707	1.07 × 10^−174^	1	246	1	246	224
XP_037203386.1 secreted in xylem 6 *Fusarium tjaetaba*	475.707	1.07 × 10^−174^	1	246	1	246	224
KAF5565621.1 secreted in xylem 6 *Fusarium napiforme*	473.781	4.55 × 10^−174^	1	246	1	246	223
KAF5589364.1 secreted in xylem 6 *Fusarium pseudocircinatum*	470.315	1.08 × 10^−172^	1	246	1	246	223
KAF5661873.1 secreted in xylem 6 *Fusarium circinatum*	466.463	3.68 × 10^−171^	9	246	1	238	221
KAF5538596.1 secreted in xylem 6 *Fusarium phyllophilum*	444.121	1.90 × 10^−162^	9	246	1	238	210
KAF5719947.1 secreted in xylem 6 *Fusarium mundagurra*	423.32	4.06 × 10^−154^	1	246	1	242	200
KAF5588511.1 secreted in xylem 6 *Fusarium pseudoanthophilum*	403.675	2.64 × 10^−146^	1	246	1	242	203
KAF5973041.1 Secreted in xylem 6 *Fusarium coicis*	348.977	3.92 × 10^−125^	1	246	1	208	180

## Data Availability

Data are available upon reasonable request.

## Supplementary Materials

The following supporting information can be downloaded at: https://www.mdpi.com/article/10.3390/jof9040443/s1, Figure S1: Multiple sequence alignment of *SIX6* genes.
