# Supplementary material for: Fusarium mindanaoense sp. nov., a New Fusarium Wilt Pathogen of Cavendish Banana from the Philippines Belonging to the F. fujikuroi Species Complex
Source: J Fungi (Basel). 2023 Apr 5;9(4):443. doi: 10.3390/jof9040443 (PMC10142649; doi:10.3390/jof9040443)
Supplement: Supplementary file 1 [file jof-09-00443-s001.zip › jof-2256677-supplementary.pdf]

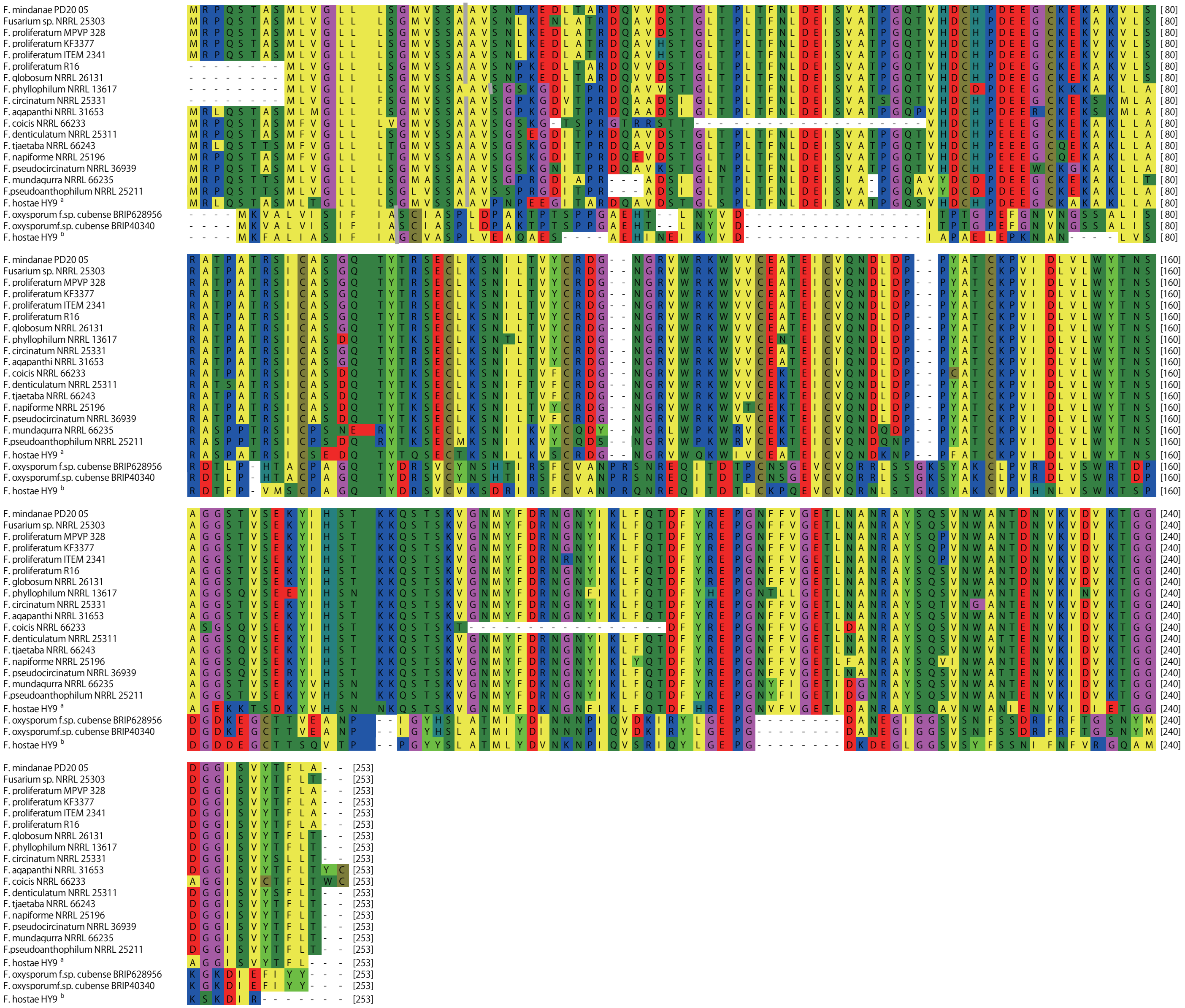

Figure S1. Multiple sequence alignment of *SIX6* genes. Two sequences of F. hostae were obtained from the genome data by tBLASTn using sequence of *F. mindanaoense* (a; PD20-05, ACY39286) and *Fusarium oxysporum* f. sp. *lycopersici* (b; BFOL51, ACY39286) as queries. The predicted SIX6 of *F. mindanaoense* (PD20-05) is shown as a representative sequence. The hyphen means gap site. Predicted cleavage sites of signal peptides are indicated by vertical bars. The alignment was conducted by ClustalW implemented in MEGA7.
